# MYC transcription factors coordinate tryptophan‐dependent defence responses and compromise seed yield in Arabidopsis

**DOI:** 10.1111/nph.18293

**Published:** 2022-06-21

**Authors:** Qiang Guo, Ian T. Major, George Kapali, Gregg A. Howe

**Affiliations:** ^1^ DOE Plant Research Laboratory Michigan State University East Lansing MI 48824 USA; ^2^ Plant Resilience Institute Michigan State University East Lansing MI 48824 USA; ^3^ Department of Biochemistry and Molecular Biology Michigan State University East Lansing MI 48824 USA

**Keywords:** amino acid metabolism, endoplasmic reticulum (ER) body, glucosinolate, growth–defence trade‐offs, jasmonate (JA), plant–insect interactions, seed yield, secondary metabolism

## Abstract

- Robust plant immunity negatively affects other fitness traits, including growth and seed production. Jasmonate (JA) confers broad‐spectrum protection against plant consumers by stimulating the degradation of JASMONATE ZIM‐DOMAIN (JAZ) proteins, which in turn relieves repression on transcription factors (TFs) coincident with reduced growth and fecundity. The molecular mechanisms underlying JA‐mediated decreases in fitness remain largely unknown.
- To assess the contribution of MYC TFs to growth and reproductive fitness at high levels of defence, we mutated three *MYC* genes in a JAZ‐deficient mutant (*jazD*) of *Arabidopsis thaliana* that exhibits strong defence and low seed yield.
- Genetic epistasis analysis showed that de‐repression of MYC TFs in *jazD* not only conferred strong resistance to insect herbivory but also reduced shoot and root growth, fruit size and seed yield. We also provided evidence that the JAZ–MYC module coordinates the supply of tryptophan with the production of indole glucosinolates and the proliferation of endoplasmic reticulum bodies that metabolise glucosinolates through the action of β‐glucosidases.
- Our results establish MYCs as major regulators of growth‐ and reproductive–defence trade‐offs and further indicate that these factors coordinate tryptophan availability with the production of amino acid‐derived defence compounds.

## Introduction

Meeting the future demand for food in an era of climate instability will require the development of high yielding crop varieties that effectively combat pathogens and herbivores (Kim *et al*., 2021). A potential impediment to achieving this goal is the frequent occurrence of trade‐offs between plant immunity and reproductive performance or growth (Smedegaard‐Petersen & Tolstrup, 1985; Karasov *et al*., 2017; Zust & Agrawal, 2017; Guo *et al*., 2018a). High levels of plant resistance to biotic stress can restrict biomass accretion and seed yield (Smedegaard‐Petersen & Tolstrup, 1985; Baldwin, 1998; Tian *et al*., 2003; Lozano‐Durán & Zipfel, 2015; Ning *et al*., 2017). Conversely, breeding for increased yield in the absence of pest pressure can compromise immunity and, in some cases, lead to crop failure from disease (Van Der Plank, 1963; Brown & Rant, 2013). A better understanding of the mechanisms that underlie the antagonistic relationships between protection and growth or yield may inform strategies to develop high yielding crops that maintain robust resistance to biotic attacks (Ning *et al*., 2017; Guo *et al*., 2018a).

Inducible production of plant defence compounds is tightly controlled by hormonal cues (Howe & Jander, 2008; Bari & Jones, 2009; Pieterse *et al*., 2012). Among the most important of these signals is the wound hormone jasmonate (JA), which both promotes defence and restricts growth (Kessler *et al*., 2004; Koo & Howe, 2009; Wasternack & Hause, 2013; Campos *et al*., 2014). In cells containing low JA levels, JA‐responsive genes are silenced by JAZ repressor proteins that bind to and inhibit various transcription factors (TFs), including the partially redundant MYC2, MYC3 and MYC4 bHLH‐type proteins (Chini *et al*., 2007; Dombrecht *et al*., 2007; Thines *et al*., 2007; Fernández‐Calvo *et al*., 2011; Niu *et al*., 2011; Qi *et al*., 2015). In response to stress‐induced accumulation of JA, JAZ proteins associate with the CORONATINE INSENSITIVE 1 (COI1)‐containing Skp1/Cullin/F‐box (SCF^COI1^) E3 ubiquitin ligase complex in a hormone‐dependent manner (Xie *et al*., 1998; Chini *et al*., 2007; Thines *et al*., 2007; Katsir *et al*., 2008). Subsequent degradation of JAZ by the 26S proteasome releases MYC TFs from repression, leading to rapid induction of defence gene expression concomitant with the downregulation of growth rate (Yan *et al*., 2007; Attaran *et al*., 2014; Havko *et al*., 2016).

Genetic analysis of the 13‐member *JAZ* family in Arabidopsis indicated that JAZs act additively not only to restrain defence responses but also to promote growth and reproductive vigour (Thireault *et al*., 2015; Campos *et al*., 2016; Guo *et al*., 2018b; Liu *et al*., 2021). For example, a moderately defended *jaz* quintuple (*jazQ*) mutant lacking *JAZ1/3/4/9/10* exhibits near‐normal seed yield and mildly reduced leaf biomass (Campos *et al*., 2016; Guo *et al*., 2018b; Major *et al*., 2020). By comparison, the strongly defended *jaz* decuple (*jazD*) mutant defective in *JAZ1/2/3/4/5/6/7/9/10/13* grows much more slowly than *jazQ* and produces less than half of the amount of seed. Genetic suppressor screens further demonstrated that, whereas the moderate growth defect in *jazQ* is dependent on the red light receptor phytochrome B (phyB), factors other than phyB mediate the strong growth inhibition in *jazD* (Major *et al*., 2020). These findings highlight the importance of defence level in understanding JA‐associated trade‐offs and may help to explain why costs of resistance are not observed under some experimental conditions (Bergelson & Purrington, 1996; Zust & Agrawal, 2017).

The ability of exogenous JA to compromise reproductive output supports the notion that induction of plant defence carries fitness costs (Baldwin, 1998; Baldwin *et al*., 1998; Agrawal *et al*., 1999; Redman *et al*., 2001; Bustos‐Segura *et al*., 2021). These observations are consistent with genetic evidence showing that the mutation of 10 *JAZ*s in Arabidopsis, or loss of the single *JAZ* gene in the liverwort *Marchantia polymorpha*, compromises reproductive success (Guo *et al*., 2018b; Howe & Yoshida, 2019; Monte *et al*., 2019). At present, little information is known about how JA‐mediated transcriptional changes reduce fitness. The capacity of MYCs to repress cell division in the seed integument (Gao *et al*., 2016; Hu *et al*., 2021) raises the possibility that these TFs contribute to losses in seed yield under conditions of high defence. Because seed and fruit production is not affected by moderate de‐repression of JA responses in *jazQ* plants, the potential role of JAZ–MYC interactions in controlling defence‐reproductive trade‐offs could not be assessed in this genetic background and therefore remains unknown (Major *et al*., 2017; Guo *et al*., 2018b).

Molecular analyses indicate that the growth–defence balance is controlled by signalling networks that, upon sensing external (e.g. light) and internal (e.g. hormones) cues, reprogramme cellular processes at the level of transcription and translation (Kliebenstein, 2016; Guo *et al*., 2018b; Ballaré & Austin, 2019; Monson *et al*., 2021). The acclimatory response hypothesis posits that downward adjustment of the growth rate in response to stress is triggered by shifts in the availability of carbon, nitrogen or other primary metabolites that are variably partitioned between growth, defence and other physiological tasks (Smith & Stitt, 2007; Guo *et al*., 2018b). We recently reported that the slow growth and strong anti‐insect defence of *jazD* plants is tightly correlated with the upregulation of the shikimate and the adjoining tryptophan biosynthetic pathways (Major *et al*., 2020). In Arabidopsis, tryptophan is a precursor of several defence‐related compounds including indolic glucosinolates, camalexin and indole‐3‐carbonylnitriles (Bednarek *et al*., 2009; Clay *et al*., 2009; Nakano *et al*., 2014; Rajniak *et al*., 2015). Tryptophan and other proteinogenic amino acids are also required to produce defensive proteins and a plethora of enzymes involved in the biosynthesis and detoxification of secondary metabolites. The strict dependence of growth and defence processes on amino acids raises the question of how partitioning of these primary metabolites is controlled during growth‐to‐defence transitions.

Multiomics analysis of *jazD* leaves showed that JAZ proteins negatively regulate the expression of many defence‐related leaf proteins, including the components of endoplasmic reticulum (ER) bodies (Guo *et al*., 2018b). These subcellular structures constitute a single‐cell defence system in which ER membrane‐encapsulated β‐glucosidases, such as BGLU23/PYK10 and BGLU18, hydrolyse indolic glucosinolates and other glucoside substrates to produce toxic compounds upon tissue damage (Nakano *et al*., 2014, 2017; Nakazaki *et al*., 2019a; Yamada *et al*., 2020; Rufian *et al*., 2021). Recent studies have indicated that the induction of ER body biogenesis is controlled by both a JA‐dependent pathway involving COI1 and MYC TFs, as well as a JA‐independent pathway involving a distinct bHLH‐type TF, NAI1 (Matsushima *et al*., 2004; Stefanik *et al*., 2019; Nakazaki *et al*., 2019b). The extent to which JAZ–MYC interactions control ER body biogenesis remains to be determined.

In this study, we investigated the contribution of MYC TFs to growth and reproductive phenotypes at high levels of defence in Arabidopsis. Genetic epistasis analyses performed with a *jazD mycT* tredecuple mutant showed that MYC TFs not only confer strong resistance to insect herbivory but also reduce fruit size, seed mass and seed yield. These findings demonstrated that the negative pleiotropic effects of JA‐triggered immunity result largely from unrestrained MYC activity. In leaves, we showed that the JAZ–MYC module co‐regulates tryptophan metabolism with the expression of BGLU18, the production of indolic glucosinolates and formation of ER bodies. Based on these data, we propose that the JAZ–MYC module plays a critical role in coordinating primary and secondary metabolism during growth‐to‐defence transitions.

## Materials and Methods

### Plant material and growth conditions

*Arabidopsis thaliana* ecotype Columbia‐0 (Col‐0) was the wild‐type (WT) genetic background for all experiments. Soil‐grown plants were maintained at 20°C (±1°C) in a growth chamber under 16 h : 8 h, day : night conditions (100 μmol m^−2^ s^−1^) unless otherwise noted. For experiments involving the growth of seedlings on agar plates, seeds were surface sterilised in 50% (v/v) bleach, washed eight times with sterile water and then stratified at least 2 d at 4°C in darkness. Seeds were then sown on 0.7% (w/v) phytoblend agar medium (Caisson Labs, Smithfield, UT, USA) containing half‐strength Linsmaier & Skoog (LS; Caisson Labs) salts supplemented with 0.8% (w/v) sucrose. Details of the mutant alleles used for the construction of the *jazD* decuple and *jazQ mycT* octuple mutants are listed in Supporting Information Table S1 and have been described previously (Major *et al*., 2017; Guo *et al*., 2018b). The *jazD mycT* tredecuple mutant was generated using the breeding and selection scheme shown in Fig. S1. PCR‐based genotyping of mutants was performed using primer sets flanking T‐DNA insertion sites, together with a third primer specific for the T‐DNA border (Campos *et al*., 2016; Major *et al*., 2017). The sequences of PCR primers used for genotyping are provided in Table S2.

### Root and shoot growth measurements

Root growth assays were performed by growing seedlings on square Petri plates (Thermo Fisher Scientific, Waltham, MA, USA) containing half‐strength LS salts, 0.8% (w/v) sucrose, and 0.7% (w/v) phytoblend agar (Shyu *et al*., 2012). Root growth assays were also performed with seedlings grown on the same medium supplemented with 15 μm 5‐methyl‐tryptophan (5‐MT; dissolved in 0.5 M HCl; Sigma‐Aldrich). Plates were incubated vertically in a growth chamber maintained at 20°C under a 16 h : 8 h, day : night (80 μmol m^−2^ s^−1^) photoperiod for 10 d. Primary root length was determined using ImageJ software (https://imagej.nih.gov/ij/). Wild‐type and mutant lines were grown on the same plate to avoid plate‐to‐plate variation. Measurement of leaf fresh weight was performed as described previously (Campos *et al*., 2016).

### Seed yield measurements

Seed yield measurements were performed essentially as described by Guo *et al*. (2018b). Briefly, an inverted plastic cone and plastic tube (Arasystem 360 kit; Arasystem, Gent, Belgium) were fitted to each plant 23 d after seed sowing. These devices allowed the collection of all seeds from dehiscing siliques at later stages of growth. Plants were well watered until flowering was complete (no new flower production), after which water was withheld for 4 wk to allow the inflorescences to dry completely. Seeds were collected from individual plants and dried with Drierite desiccant for 2 wk, after which the total seed mass per plant was measured. Average seed mass was determined by weighing dry seeds in batches of 100. For each plant, the weights of five sample batches were measured and averaged. The silique length and number of seeds per silique were determined by sampling the fully elongated seventh, ninth and eleventh siliques on the main stem.

### Insect‐feeding assays

Insect‐feeding assays were performed with soil‐grown plants maintained at 20°C in a growth chamber with a photoperiod of 8 h : 16 h, light : dark (100 μmol m^−2^ s^−1^). Three neonatal *Trichoplusia ni* larvae (Benzon Research) were reared on each of 11 plants (10‐wk‐old) for 10 d, after which larval weights were measured (Herde *et al*., 2013). Feeding trials were terminated several days before complete removal of leaf tissue by insect larvae.

### Quantification of glucosinolate and tryptophan levels

Glucosinolate measurements were performed using 23‐d‐old plants grown under long‐day conditions (16 h : 8 h, day : night). For measurement of glucosinolates, rosette leaves were harvested and immediately frozen in liquid nitrogen. Target compounds were extracted with 80% methanol as described previously (Glauser *et al*., 2012). Samples were analysed using a Waters (Milford, MA, USA) Xevo G2‐XS ultrahigh pressure liquid chromatography (UPLC) system coupled to quadrupole time‐of‐flight mass spectrometry (QTOF‐MS) in the MSU Mass Spectrometry Facility. Glucosinolates were processed in negative mode. Data analysis and processing were performed as described previously (Glauser *et al*., 2012). Whole rosettes from long‐day grown plants were harvested and used for quantification of tryptophan levels. Amino acids were extracted with Milli‐Q water containing labelled standards (^13^C,^15^N‐labelled amino acids; Sigma‐Aldrich). Extracts were incubated at 90°C for 5 min and cooled on ice. The extracts were clarified by centrifugation and filtered through low‐binding hydrophilic polytetrafluoroethylene (PTFE) filters (0.2 μM; Millipore). Filtered extracts were diluted with 20 mM perfluoroheptanoic acid (PFHA; Sigma‐Aldrich) and analysed using a Quattro Micro API LC–MS/MS (Waters) equipped with an Acquity 623 UPLC HSS T3 1.8 μm column (2.1 × 100 mm; 1.8 μm particle size; Waters) as described previously (Major *et al*., 2020). Tryptophan concentrations were determined from an external standard curve and normalised to tissue weight.

### Protein analysis

Proteins were extracted from leaves of 23‐d‐old soil‐grown plants with a buffer containing 100 mM Tris‐HCl pH 6.8, 150 mM NaCl, 10% glycerol, 4% sodium dodecyl sulphate (SDS), 200 mM dithiothreitol (DTT), and one tablet of Complete Mini ethylene diamine tetraacetic acid (EDTA)‐free proteinase inhibitors (one tablet per 10 ml; Roche). Lipophilic contaminants were removed by chloroform–methanol extraction of the solubilised protein. Following chloroform–methanol extraction, the protein pellet was resuspended in a buffer containing 100 mM Tris‐HCl pH 6.8, 150 mM NaCl, 10% glycerol, 1% SDS, and 200 mM DTT. Extracted proteins were separated by SDS–polyacrylamide gel electrophoresis (SDS‐PAGE) and stained with Coomassie Brilliant Blue R‐250. The *jazD*‐specific band (*c*. 65 kDa region of the gel) was excised and digested in‐gel following the protocol of Shevchenko *et al*. (1996) with minor modifications. Briefly, proteins within the gel were digested by trypsin and peptides were extracted from the gel by water bath sonication in a solution of 60% acetonitrile acidified with 1% TCA. Peptides were sprayed into a Thermo Fisher Q‐Exactive mass spectrometer using a FlexSpray spray ion source. The resulting MS/MS spectra were converted to peak lists using Mascot Distiller, v.2.5.1 (www.matrixscience.com) and searched against a custom database containing Arabidopsis protein sequences (TAIR, v.10, downloaded from the Arabidopsis Information Network, www.arabidopsis.org) and appended with common laboratory contaminants (downloaded from www.thegpm.org, cRAP project) using the Mascot searching algorithm, v.2.5.1. The Mascot output was then analysed using Scaffold, v.4.5.0 (www.proteomesoftware.com) to probabilistically validate protein identifications. Assignments validated using the scaffold 1% FDR confidence filter were considered true.

### Quantitative PCR

For quantitative PCR (qPCR) analysis, rosette leaves of two 23‐d‐old soil‐grown plants were pooled for each sample, with three biological replicates collected per sample. The analysis was performed as previously described (Major *et al*., 2020). Briefly, and according to manufacturer's instructions, RNA was extracted using the NucleoSpin plant RNA extraction kit (Macherey‐Nagel, Allentown, PA, USA). Following quality assessment by A260/A280 ratios, RNA was reverse transcribed using the High Capacity cDNA Reverse Transcription kit (Applied Biosystems, Waltham, MA, USA). qPCR reactions were run using the Power SYBR Green Master Mix (Applied Biosystems) on an Applied Biosystems 7500 Fast qPCR instrument with a dissociation curve to confirm a single peak for each set of primers. Primer efficiencies for each primer pair were determined with LinRegPCR v.2012.0 and target gene expression was normalised to the expression of *PROTEIN PHOSPHATASE 2A* (*PP2A*).

### Confocal laser scanning microscopy

The coding sequence of ER luminal marker gene *ER–YFP* included the signal peptide of WALL‐ASSOCIATED KINASE 2 (WAK2), YFP and HDEL ER retention signal (Nelson *et al*., 2007). *ER–YFP* was subcloned into the pYL436 binary vector, which contained the Cauliflower Mosaic Virus *35S* promoter sequence. Following introduction of this binary vector construct into *Agrobacterium tumefaciens* strain GV3101, the resulting strain was used to transform WT and *jazD* plants using the floral dip method (Clough & Bent, 1998). Seedlings (T1 generation) of transformed lines were screened on LS plates containing gentamycin (100 μg ml^−1^) and resistant plants were transferred into soil. Homozygous lines were selected by testing the T3 progeny for resistance to gentamycin. A *jazD mycT* line overexpressing *ER–YFP* was obtained by crossing *jazD mycT* to *jazD* plants harbouring the *35S:ER–YFP* transgene. A *jazD mycT* line containing the transgene was confirmed by PCR genotyping to be homozygous for all three *myc* mutations. The fifth rosette leaves of 30‐d‐old homozygous lines were inspected by confocal laser scanning microscopy with a Nikon (Tokyo, Japan) A1Rsi microscope. NIS‐Elements Advanced Research (Nikon) and Photoshop (Adobe, San Jose, CA, USA) software were used for image processing.

### Leaf respiration

Daytime leaf respiration was determined as described by Major *et al*. (2020). Briefly, plants were grown in plastic containers (‘Cone‐tainers’; Steuwe and Sons, Tangent, OR, USA) under an 8 h : 16 h, day : night, 21°C : 18°C photoperiod (120 μmol m^−2^ s^−1^). Single, attached mature rosette leaves of 8‐ to 10‐wk‐old plants were assessed using the LI‐6800 system (Li‐Cor Biosciences, Lincoln, NE, USA) outfitted with a standard leaf chamber (6 cm^2^ chamber area), with area corrected to the actual measured leaf area. Leaves were acclimated for at least 20 min with 400 ppm CO_2_, 0.85 kPa leaf vapour pressure deficit, and a light intensity of 500 μmol m^−2^ s^−1^, with leaf temperature maintained at 21°C. Respiration was determined from slope–intercept regression analysis of the intersection of five CO_2_ response curves (using intercellular CO_2_ below 10 Pa) measured at decreasing, subsaturating irradiances (Walker *et al*., 2016).

### Accession numbers

Genes described here have the following Arabidopsis Genome Initiative (AGI) gene accession numbers: *JAZ1* (AT1G19180), *JAZ2* (AT1G74950), *JAZ3* (AT3G17880), *JAZ4* (AT1G48500), *JAZ5* (AT1G17380), *JAZ6* (AT1G72450), *JAZ7* (AT2G34600), *JAZ9* (AT1G70700), *JAZ10* (AT5G13220), *JAZ13* (AT3G22275), *MYC2* (AT1G32640), *MYC3* (AT5G46760), *MYC4* (AT4G17880), *COI1* (AT2G39940), *NAI1* (AT2G22770), *NAI2* (AT3G15950), *PYK10* (AT3G09260), *BGLU18* (AT1G52400), *TSA1* (AT1G52410) and *PP2A* (AT1G13320).

## Results

### MYC TFs impair growth and reproductive performance at high levels of defence

**Fig. 1:**
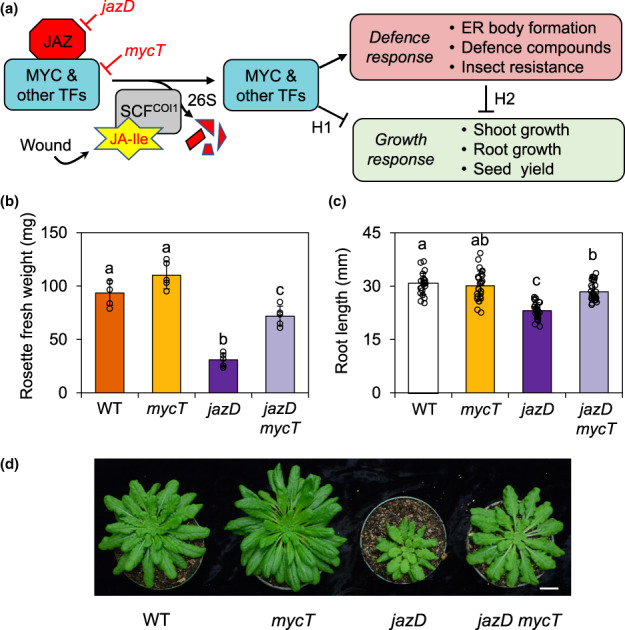
Hyperactivation of MYC transcription factors (TFs) in a *jasmonate zim‐domain* decuple mutant (*jazD*) of Arabidopsis restricts shoot and root growth. (a) Schematic of the jasmonate (JA) signalling pathway and its various growth and defence‐related outputs. JAZ‐repressible TFs (e.g. MYCs) modulate JA responses involved in growth and defence. Genetic perturbations (blunt‐ended red arrows) used in this study to manipulate JA responses include loss‐of‐function mutations in 10 genes encoding JASMONATE ZIM‐DOMAIN (JAZ) repressors (*jazD*) or three genes encoding MYC TFs (*mycT* or *myc234*). Wound‐induced accumulation of endogenous JA‐Ile results in COI1‐dependent JAZ ubiquitylation and subsequent degradation via the 26S proteasome (26S). Two general hypotheses (*H*_1_ and *H*_2_) to explain how de‐repression of TF activity inhibits (blunt‐ended black arrows) growth in the JAZ‐depleted *jazD* mutant. *H*_1_, TFs control the expression of genes that directly affect growth responses. *H*_2_, changes in metabolism resulting from TF‐dependent defence responses indirectly affect growth. (b) Rosette fresh weight of 26‐d‐old wild‐type (WT), *mycT*, *jazD* and *jazD mycT* plants. Values show the mean ± SD (*n* = 5 biological replicates). Letters denote significant differences according to Tukey's honestly significant difference (HSD) test (*P* < 0.05). (c) Root length of 10‐d‐old WT, *mycT*, *jazD* and *jazD mycT* seedlings grown on plates. Values show the mean ± SD (*n* = 24–30 biological replicates). Letters denote significant differences according to Tukey's HSD test (*P* < 0.05). (d) Photograph of representative WT, *mycT*, *jazD* and *jazD mycT* plants grown for 73 d under short‐day conditions (8 h : 16 h, day : night). Bar, 2 cm.

We used *jazD* as a model to study the transcriptional control of growth–defence and reproductive–defence trade‐offs at high levels of defence, without the need to elicit responses with exogenous JA. As illustrated in Fig. 1a, the reduced growth and reproductive fitness of *jazD* could result from de‐repression of the partially redundant MYC2/3/4 TFs or possibly other JA‐responsive factors. To distinguish between these possibilities, we used genetic crosses to introduce three loss‐of‐function *myc* mutations (*myc2‐1*, *myc3‐1* and *myc4‐1*; referred to as *mycT*) into the *jazD* background (Fig. S1) and then compared growth and defence traits in the resulting *jazD mycT* tredecuple mutant to WT, *jazD*, and *mycT* plants grown under long‐day (16 h : 8 h, day : night) conditions. Leaf biomass of *mycT* plants was consistently higher than that of WT (Fig. 1b), although the difference was not statistically significant, as previously reported (Major *et al*., 2017). The reduced rosette biomass of *jazD* (33% of WT) was largely recovered (77% of WT) by the loss of *MYC2/3/4* in the *jazD mycT* line (Fig. 1b). The rosette fresh weight of *jazD mycT* was significantly higher than that of *jazD*, but it was lower than that of WT. A similar growth recovery by *mycT* was observed in *jazD* plants maintained under short‐day conditions (8 h : 16 h, light : dark; Fig. 1d). We also tested whether MYC2/3/4 contributed to the reduced root growth of *jazD* plants. Root growth assays showed that, whereas the root length of WT and *mycT* seedlings were comparable, the short‐root phenotype of *jazD* was mostly rescued (93% of WT) by *mycT* (Fig. 1c). However, the root length of *jazD mycT* was still significantly shorter than that of WT. We did not observe obvious differences in root branching among these mutants. Consistent with the JAZ restraint model of JA signalling (Fig. 1a), these data indicated that the constitutive reduction of shoot and root growth in *jazD* plants is mediated in large part by the action of MYC2/3/4.

**Fig. 2:**
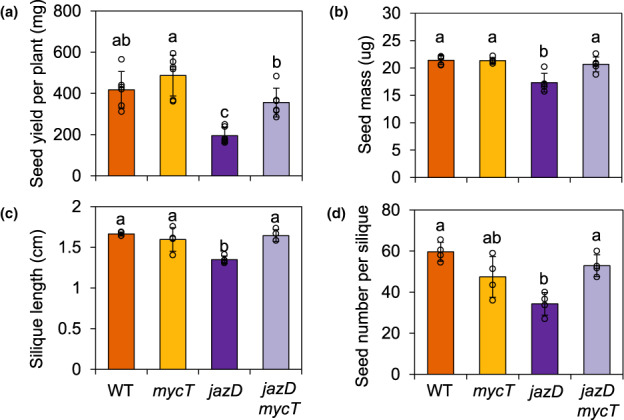
MYC transcription factors restrict seed production in a *jasmonate zim‐domain* decuple mutant (*jazD*) of Arabidopsis. (a, b) Seed yield (a) and individual seed mass (b) of wild‐type (WT), *mycT*, *jazD* and *jazD mycT* plants. Values show the mean ± SD (*n* = 6 biological replicates). (c, d) Silique length (c) and seed number per silique (d) of WT, *mycT*, *jazD* and *jazD mycT* plants. Data were obtained from the fully elongated seventh, ninth and eleventh siliques of the main inflorescence. Values are means, with bars showing SD, of four plants per genotype. In all panels, letters denote significant differences according to Tukey's honestly significant difference (HSD) test (*P* < 0.05).

We next investigated how genetic interactions between *jazD* and *mycT* affected reproductive success. Previous studies showed that the seed yield of *jazD* plants is less than half that of WT and that seed size is also reduced in this mutant (Guo *et al*., 2018b). Markedly, these fitness penalties in *jazD* were completely rescued by the addition of *mycT* (Fig. 2a,b). The seed yield and seed size of *jazD mycT* were not statistically different from those of WT. We also found that the reduced silique length and seed number per fruit of *jazD* were restored to near WT levels in the *jazD mycT* combinatorial mutant (Fig. 2c,d). No significant difference was found in silique length and seed number between WT and *jazD mycT*. These data demonstrate that, in the absence of environmentally imposed stress, loss of JAZ‐mediated restraint on MYC2/3/4 compromised multiple aspects of reproductive success to curtail seed yield.

**Fig. 3:**
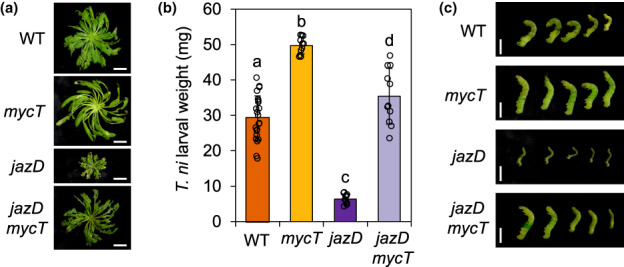
MYC transcription factors promote strong resistance of the *jasmonate zim‐domain* decuple mutant (*jazD*) of Arabidopsis to insect herbivory. (a) Photograph of representative short‐day grown wild‐type (WT), *mycT*, *jazD* and *jazD mycT* plants after challenge with three *Trichoplusia ni* larvae for 10 d. Bars, 1 cm. (b) Weight of larvae reared on the indicated genotypes. Values show the mean ± SD of at least 30 larvae per host genotype. Letters denote significant differences according to Tukey's honestly significant difference (HSD) test (*P* < 0.05). (c) Photograph of representative *T. ni* larvae grown on the indicated host genotype. Bar, 0.5 cm.

We used insect bioassays to determine whether the negative impact of MYCs on the growth and fitness of *jazD* is linked to the ability of these TFs to promote high levels of leaf protection against herbivory as indicated in Fig. 1a. Results from the insect‐feeding trials showed that the weight gain of *Trichoplusia ni* (cabbage looper) larvae reared on *mycT* plants was greater than that on WT, whereas larvae performed very poorly on *jazD* plants (Fig. 3). Insect performance on the *jazD mycT* combinatorial mutant was intermediate between that of larvae grown on WT and *mycT* plants, indicating that the strong resistance of *jazD* is largely dependent on MYC2/3/4. These collective data are consistent with previous studies showing that MYC TFs play a major role in promoting resistance of WT and *jazQ* plants to generalist herbivores (Fernández‐Calvo *et al*., 2011; Schweizer *et al*., 2013; Major *et al*., 2017; Guo *et al*., 2018b), and further indicated that unrestrained MYC activity promotes strong anti‐insect defence, concomitant with the onset of growth and reproductive penalties.

### JAZ depletion triggers ER body formation via a MYC‐dependent pathway

**Fig. 4:**
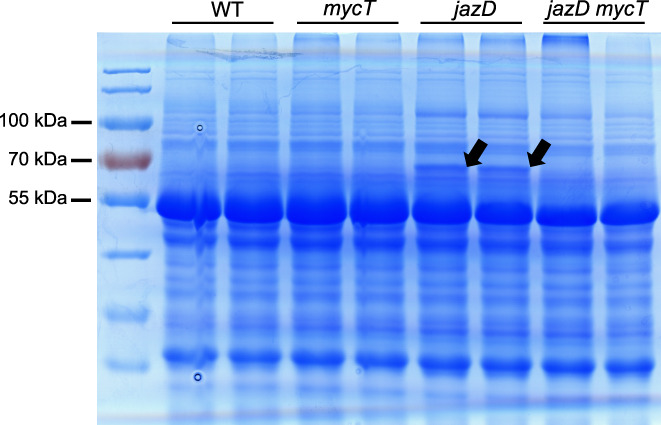
Overaccumulation of β‐glucosidase18 (BGLU18) in leaves of the *jasmonate zim‐domain* decuple mutant (*jazD*) of Arabidopsis is dependent on MYC transcription factors. Photograph of a Coomassie blue‐stained sodium dodecyl sulphate–polyacrylamide gel electrophoresis (SDS‐PAGE) gel of total protein from leaves of the indicated genotype. Duplicate samples (biological replicates) were loaded for each genotype. Arrows denote a *c*. 65‐kDa protein band that corresponds to BGLU18 and is specific to *jazD*. Protein standards and their corresponding mass (kDa) are shown on the left.

**Fig. 5:**
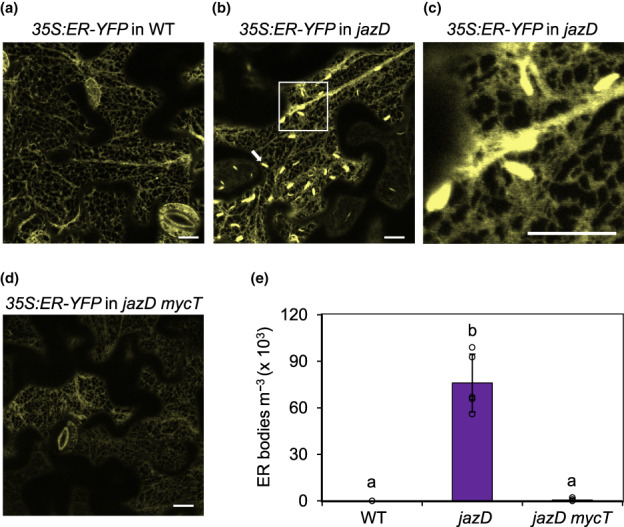
Constitutive endoplasmic reticulum (ER) body formation in leaves of the Arabidopsis *jasmonate zim‐domain* decuple mutant (*jazD*) requires MYC transcription factors. (a–d) Confocal images (a, b, d) showing the ER in leaves (5^th^ oldest rosette leaf) of 24‐d‐old wild‐type (WT), *jazD* and *jazD mycT* plants expressing an ER luminal marker protein, ER–YFP. (c) Enlargement of the highlighted region shown in panel (b). The white arrow depicts an ER structure. Bar, 10 μm. (e) Quantification of ER body number in WT, *jazD* and *jazD mycT* leaves. ER bodies were counted in confocal z‐stack images and expressed per unit volume. Values show the mean ± SD (*n* = 5 biological replicates). Lowercase letters denote significant differences according to Tukey's honestly significant difference (HSD) test (*P* < 0.05).

Plant resistance to herbivores and pathogens often depends on the induced accumulation of leaf defence compounds. To test whether the strong growth and defence phenotypes of *jazD* plants are associated with changes in the accumulation of highly abundant proteins, we used SDS‐PAGE to profile the bulk protein content of WT, *mycT* and *jazD mycT* leaves. The results showed that the protein profile of all genotypes was qualitatively similar with the obvious exception of a *c*. 65‐kDa protein band that was specific to *jazD* (Fig. 4). Mass spectrometry analysis showed that the major polypeptide in this band was β‐glucosidase 18 (BGLU18; encoded by AT1G52400), with lesser amounts of NADP‐malic enzyme 2 (ME2; encoded by AT5G11670) also detected (Fig. S2). BGLU18 is a major component of ER bodies, which proliferate in leaves in response to wounding or JA treatment (Ogasawara *et al*., 2009). To test whether JAZ–MYC interactions controlled the formation of ER body structures in leaves, a transgene (*35S::ER–YFP*) encoding a YFP‐tagged ER marker protein (ER–YFP) was stably transformed onto WT, *jazD* and *jazD mycT* genetic backgrounds. Confocal microscopy of leaf tissue from the resulting lines showed that fusiform structures indicative of ER bodies were present in ER–YFP‐expressing *jazD* leaves, but not in WT or *jazD mycT* leaves (Fig. 5a–d). Quantification of ER body number showed that the formation of ER bodies in *jazD* was strictly dependent on MYC2/3/4 (Fig. 5e). We concluded that loss of JAZ‐mediated repression in *jazD* was sufficient to trigger the formation of leaf ER bodies via a pathway that depends on MYC TFs.

**Fig. 6:**
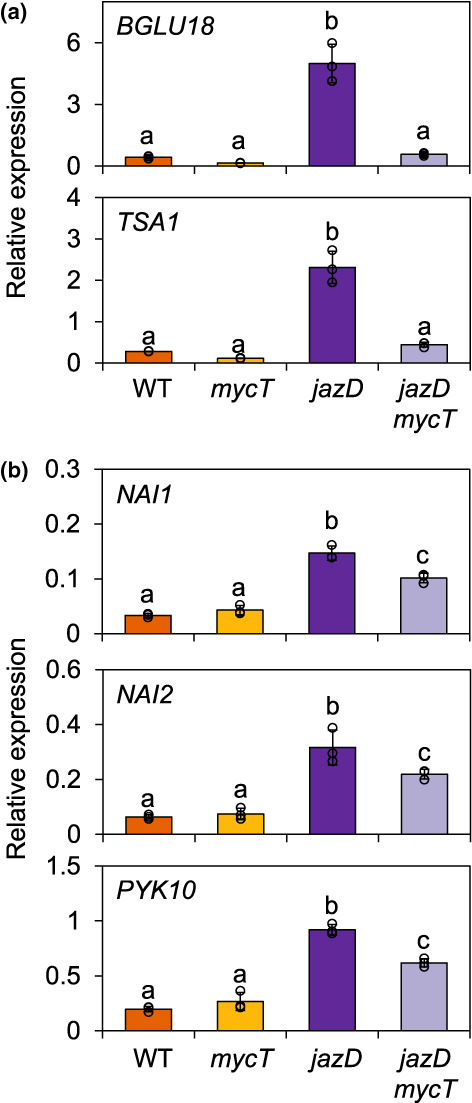
Induction of endoplasmic reticulum (ER) body genes in leaves of the Arabidopsis *jasmonate zim‐domain* decuple mutant (*jazD*) is mediated by MYC2/3/4. (a) Relative expression levels of genes encoding components of inducible ER bodies. *β‐glucosidase18* (*BGLU18*) and *tonsoku‐associating protein 1* (*TSA1*) were used as marker genes. (b) Relative expression levels of genes encoding components of constitutive ER bodies. *NAI1*, *NAI2* and *PYK10* were used as marker genes. For marker genes shown in both panels, mRNA was isolated from the leaves of 26‐d‐old wild‐type (WT), *mycT*, *jazD* and *jazD mycT* plants. Expression levels were normalised to the reference gene *PP2a* and show the mean ± SD (*n* = 3 biological replicates). Lowercase letters denote significant differences according to Tukey's honestly significant difference (HSD) test (*P* < 0.05).

ER bodies in Arabidopsis are constitutively expressed in seedlings (cER bodies) or induced by the JA pathway in leaves (iER bodies) (Nakano *et al*., 2014). To better define the role of the JAZ–MYC module in controlling ER body biogenesis, we used quantitative reverse transcription (qRT)‐PCR to measure the abundance of mRNAs encoding proteins that are preferentially associated with cER and iER bodies. We found that *jazD* leaves accumulated high levels of *BGLU18* mRNA together with *TSA1* transcripts, which encoded an iER body‐specific protein regulated by the JA pathway (Geem *et al*., 2019; Stefanik *et al*., 2019). Consistent with this finding, the high constitutive expression of *BGLU18* and *TSA1* in *jazD* leaves was restored to WT levels in the *jazD mycT* combinatorial line (Fig. 6a). Genes encoding cER‐specific components, including NAI1 (a bHLH TF), NAI2 (a homologue of TSA1) and PYK10/BGLU23, were also induced in *jazD*, albeit to lower levels than iER body‐specific genes (Fig. 6b). We also found that the expression of cER body genes in *jazD* was only marginally reduced by *mycT*. These data indicated that, whereas JAZ deficiency is sufficient to activate the expression of genes encoding components of both cER and iER bodies, the JAZ–MYC module exerts stronger control over the expression of iER body compared with cER body genes.

### Coordinated regulation of indole glucosinolate and tryptophan metabolism by the JAZ–MYC module

**Fig. 7:**
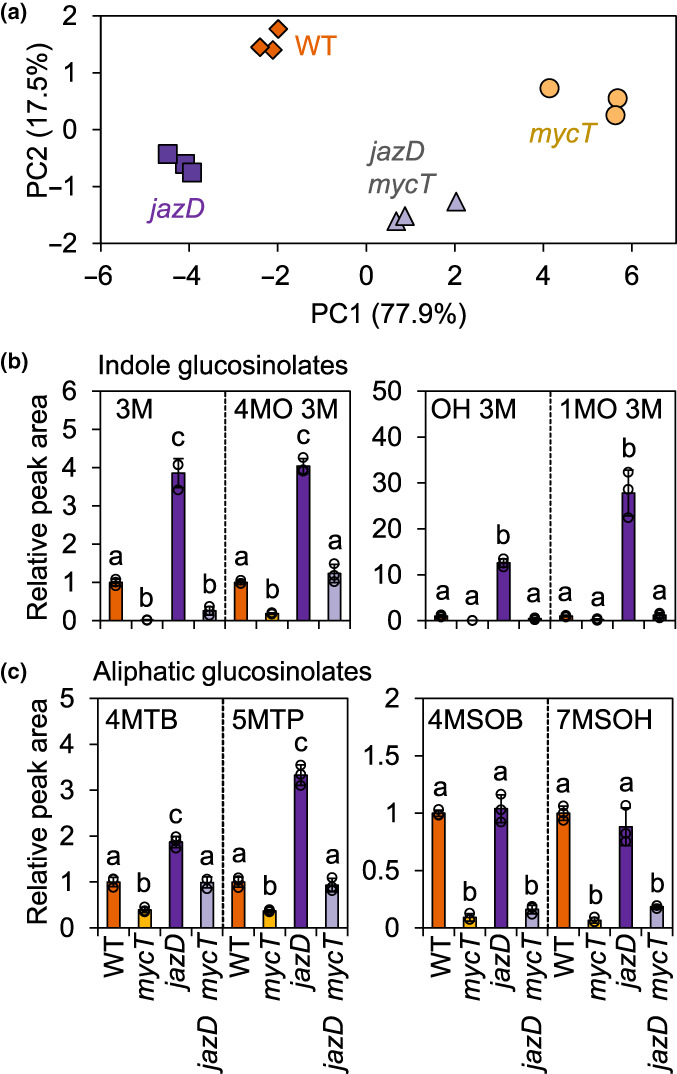
Constitutive accumulation of glucosinolates in the *jasmonate zim‐domain* decuple mutant (*jazD*) of Arabidopsis is dependent on MYC transcription factors. (a) Principal component analysis (PCA) of leaf glucosinolate levels in wild‐type (WT), *mycT*, *jazD* and *jazD mycT* plants. Each point shows the total glucosinolate content (indolic and aliphatic) of a single plant. (b, c) Indole (b) and aliphatic (c) glucosinolate levels in leaves of 26‐d‐old WT, *mycT*, *jazD* and *jazD mycT* plants. Values show the mean ± SD of three biological replicates. The peak area from liquid chromatography–mass spectrometry (LC–MS) for the indicated compound in the WT sample was set to ‘1’ and the peak area of the same compound in other genotypes was scaled to the WT sample. Letters denote significant differences according to Tukey's honestly significant difference (HSD) test (*P* < 0.05). I3M, OHI3M, 4MOI3M and 1MOI3M (b) belong to indole glucosinolates: I3M, indol‐3‐ylmethyl (glucobrassicin); OHI3M, 4‐hydroxyindol‐3‐ylmethyl (hydroxyglucobrassicin); 4MOI3M, 4‐methoxyindol‐3‐ylmethyl (methoxyglucobrassicin); 1MOI3M, 1‐methoxyindol‐3‐ylmethyl (neoglucobrassicin). 4MTB, 5MTP, 4MSOB and 7MSOH (c) belong to aliphatic glucosinolates: 4MTB, 4‐methylthiobutyl (glucoerucin); 5MTP, 5‐methylthiopentyl (glucoberteroin); 4MSOB, 4‐methylsulphinylbutyl (glucoraphanin); 7MSOH, 7‐methylsulphinylheptyl (glucoibarin).

As a single‐cell defence system, the efficacy of ER body‐based immunity depends on the coordinated expression of ER body structural components, BGLU hydrolases and glucosinolate substrates (Yamada *et al*., 2020). Accordingly, we assessed the role of the JAZ–MYC module in controlling glucosinolate accumulation in leaves. LC–MS analysis of leaf extracts from untreated plants showed that the total glucosinolate profile of WT, *mycT*, *jazD* and *jazD mycT* was highly distinct based on principal component analysis (PCA), which explained *c*. 95% of the glucosinolate variance between genotypes (Fig. 7a). The level of both aliphatic and indolic glucosinolates was strongly reduced in *mycT* relative to WT (Figs 7b,c, S3), confirming the findings of previous studies (Schweizer *et al*., 2013). *jazD* leaves accumulated increased levels of nearly all indolic glucosinolate derivatives. Moreover, the stimulatory effect of JAZ depletion on indolic glucosinolate production was reversed by *mycT* (Fig. 7b). A similar pattern was observed for a subset of aliphatic glucosinolates, including 4MTB, 5MTP, 5MSOP, and 7MTH (Figs 7c, S3). We noted, however, that the accumulation of other aliphatic derivatives, including 4MSOB, 7MSOH, 3MSOP, 6MSOH, 3MTP and 8MSOO, was not elevated in *jazD*, but remained strongly attenuated in *mycT* and *jazD mycT* leaves (Figs 7c, S3). These patterns of glucosinolate accumulation are consistent with current models, indicating that MYCs, in conjunction with MYB TFs, contributed to both basal and JA‐inducible glucosinolate accumulation (Schweizer *et al*., 2013).

**Fig. 8:**
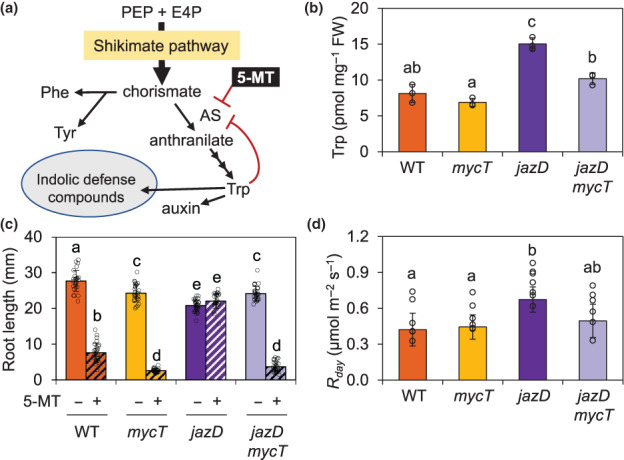
Increased tryptophan metabolism in the *jasmonate zim‐domain* decuple mutant (*jazD*) of Arabidopsis is dependent on MYC transcription factors. (a) Simple schematic of aromatic amino biosynthesis and the partitioning of tryptophan (Trp) to the production of indole defence compounds. Anthranilate synthase (AS) catalyses the first step in the tryptophan branch of aromatic amino acid biosynthesis and is feedback inhibited (blunt‐ended red arrows) by tryptophan and the toxic tryptophan analogue 5‐methyl‐tryptophan (5‐MT). PEP, phosphoenolpyruvic acid; E4P, erythrose 4‐phosphate. (b) Tryptophan levels in leaves of 21‐d‐old wild‐type (WT), *mycT*, *jazD* and *jazD mycT* plants. Values show the mean ± SD (*n* = 3 biological replicates). Lowercase letters denote significant differences according to Tukey's honestly significant difference (HSD) test (*P* < 0.05). (c) Root length of 10‐d‐old WT, *mycT*, *jazD* and *jazD mycT* seedlings grown on plates supplemented (hatched) or not supplemented (open) with 15 μM 5‐MT. Values show the mean ± SD (*n* = 26–29 biological replicates). Lowercase letters denote significant differences according to Tukey's HSD test (*P* < 0.05). (d) Leaf respiration rates of 10‐wk‐old WT, *mycT*, *jazD* and *jazD mycT* plants measured in mature leaves by gas exchange. Respiration was determined from the common intersection of CO_2_ response curves measured under subsaturating irradiances. Values show the mean ± SD (*n* = 6 or 7 biological replicates). Lowercase letters denote significant differences according to Tukey's HSD test (*P* < 0.05).

The biosynthesis of indolic glucosinolates and other indolic defence compounds from tryptophan raised the possibility that JAZs and MYCs functionally interacted to coordinate secondary metabolism with the supply of tryptophan generated through primary metabolism (Fig. 8a). Indeed, we found that *jazD* leaves contained significantly higher levels of tryptophan than WT and that this stimulatory effect was dependent on MYC TFs (Fig. 8b). As an independent measure of the rate of tryptophan metabolism, we determined the sensitivity of WT, *mycT*, *jazD*, and *jazD mycT* seedlings to 5‐methyl‐tryptophan (5‐MT). Both tryptophan and 5‐MT exerted feedback inhibition on anthranilate synthase (AS), which performs the rate‐limiting step in the tryptophan biosynthetic pathway (Fig. 8a). Unlike tryptophan, however, 5‐MT does not support protein synthesis and therefore impedes growth at concentrations that inhibit AS (Celenza *et al*., 2005). Previous studies have shown that the root growth of *jazD* is strongly insensitive to 15 μM 5‐MT, indicative of increased expression of AS and other tryptophan biosynthetic enzymes (Guo *et al*., 2018b; Major *et al*., 2020). As shown in Fig. 8c, *mycT* abolished the insensitivity of *jazD* to 5‐MT and also increased 5‐MT sensitivity in an otherwise WT (i.e. JAZ replete) genetic background. In demonstrating that *mycT* is epistatic to *jazD* for 5‐MT resistance, these data supported the hypothesis that MYC TFs stimulated tryptophan biosynthetic capacity under JA‐mediated stress conditions.

Consistent with the notion that the *de novo* synthesis of defence compounds involves increased rates of cellular respiration to meet the demand for energy equivalents and carbon skeletons (Bolton, 2009), *jazD* leaves exhibited higher rates of respiration than WT (Guo *et al*., 2018b; Major *et al*., 2020). This observation prompted us to test whether MYC TFs were required for the stimulatory effect of *jazD* on leaf respiration. We found that, although *mycT* alone had no effect on respiration, the loss of MYC2/3/4 in *jazD mycT* dampened the respiration rate in *jazD* leaves, although the difference was not statistically significant (Fig. 8d). These data suggested that the MYC‐dependent increase in tryptophan metabolism in *jazD* is part of a broader effect of unrestrained MYC activity on central metabolism.

## Discussion

### Hyperactive MYC TFs restrict vegetative growth

Plant growth–defence balance is influenced by multiple hormones and environmental signals. The genetic complexity underlying such phenotypic plasticity has hampered efforts to identify regulatory factors that curtail growth and reproductive fitness during active immunity. As master regulators of JA responses, MYC TFs are subject to multiple levels of transcriptional and post‐transcriptional control that potentially influence the growth–defence balance (Chini *et al*., 2007; Dombrecht *et al*., 2007; Withers *et al*., 2012; Schweizer *et al*., 2013; Zhai *et al*., 2013; An *et al*., 2017; Chico *et al*., 2020). Here, we used genetic epistasis to determine the contribution of JAZ–MYC interactions to pleiotropic phenotypes resulting from the overactivation of the JA sector of immunity. A primary conclusion of our work is that loss of JAZ‐mediated restraint on MYCs is largely responsible for downward adjustment of growth in roots and leaves of a mutant (*jazD*) that is defective in most JAZ family members. The fact that *mycT* did not fully restore these growth phenotypes of *jazD* to WT levels raises the possibility that additional JAZ‐interacting TFs, or non‐*JAZ* genetic polymorphisms resulting from the construction of *jazD*, make minor contributions to growth restriction in vegetative tissues. Nevertheless, these and other findings (Major *et al*., 2017) demonstrate the importance of MYC TFs in dampening growth across a range of defence levels in Arabidopsis. Functional characterisation of MYC TFs in the liverwort *Marchantia polymorpha* (Penuelas *et al*., 2019) indicates that the role of MYCs in growth–defence antagonism is likely to be conserved in the plant kingdom.

Mechanistic studies have demonstrated how fluctuations in bioactive jasmonate (i.e. jasmonoyl‐l‐isoleucine) levels dynamically switch MYCs between opposing states of target gene repression and activation to promote growth and defence, respectively (An *et al*., 2017; Zhang *et al*., 2017; Howe *et al*., 2018; Guo *et al*., 2018b). Despite these insights, knowledge of how MYC action restricts tissue growth is only beginning to emerge. In roots, MYC2 binds to and represses the expression of genes encoding AP2‐domain PLETHORA TFs (PLT1 and PLT2), which are required for auxin‐mediated stem cell maintenance and root elongation (Chen *et al*., 2011). Growth–defence balance in shoot tissues is controlled in part by functional interactions between JAZ and DELLA proteins, the latter of which repress gibberellin (GA)‐mediated growth responses upon JA‐induced JAZ depletion (Hou *et al*., 2010; Yang *et al*., 2012; Monson *et al*., 2021). Recent studies, however, have failed to provide evidence that changes in the abundance of RGA (a DELLA family member) or altered sensitivity to GA account for the growth reduction of *jazD* shoots (Major *et al*., 2020). We further show here that the slow growth of *jazD* can be rescued by an epistatic mutation (*mycT*) that presumably does not affect functional interactions between JAZ and DELLA. Given the capacity of MYC2 to interact with RGA (Hong *et al*., 2012), we cannot exclude the possibility that unrestrained MYC activity impacts GA‐mediated growth responses independently of DELLAs. An alternative hypothesis is that JA‐mediated shoot growth inhibition is triggered by MYC‐mediated changes in metabolism (please refer to the following sections) (Attaran *et al*., 2014; Bömer *et al*., 2018; Major *et al*., 2020; Zander *et al*., 2020).

### MYC TFs exert negative pleiotropic effects on reproductive performance

As key determinants of plant fitness, seed quality and yield are negatively impacted by genetic programmes that promote resistance to biotic attack (Karasov *et al*., 2017). We have previously reported that constitutive activation of JA signalling in *jazD* greatly reduces seed yield and that loss of one additional repressor (JAZ8) causes near‐complete loss of seed production in the resulting *jaz* undecuple mutant (Guo *et al*., 2018b). Consistent with these findings, the analysis of *jaz* single mutants implicated JAZ5 and JAZ6 in promoting increased seed size in Arabidopsis (Hu *et al*., 2021). It has also been shown that exogenous JA acts in a dose‐dependent manner to reduce the size of WT seed, most likely through the de‐repression of MYC TFs (Hu *et al*., 2021). In demonstrating that *mycT* restores the reduced seed size and low seed yield of *jazD* plants to WT levels, our results supported the view that unrestrained MYC activity is the primary cause of JA‐mediated decreases in fecundity across a range of defence levels.

The mechanisms by which MYCs reduce seed production remain to be determined but, as in other pleiotropic phenotypes, are likely to be complex. Gao *et al*. (2016) showed that MYC TFs act synergistically in Arabidopsis to reduce seed size and to modulate the accumulation of the major seed storage proteins. Similarly, Hu *et al*. (2021) demonstrated that MYCs repress cell proliferation in the seed integument. Studies showing that elicitation of leaf defence via exogenous JA compromises reproductive performance (Baldwin, 1998; Agrawal *et al*., 1999; Redman *et al*., 2001; Bustos‐Segura *et al*., 2021) have suggested that the redirection of nutrients to the biosynthesis of defence compounds may contribute to declines in reproductive fitness. For example, JA‐elicited defence reduced the availability of N for use in seed production (Baldwin *et al*., 1998). A better understanding of how the JAZ–MYC regulon controls seed yield has the potential to inform biotechnological approaches to mitigate the negative effects of JA signalling on grain production in crop plants (Dampanaboina *et al*., 2019).

### The JAZ–MYC regulon co‐regulates ER body formation and glucosinolate accumulation

JA promotes not only the biosynthesis of defence‐related metabolites but also the development of multicellular and subcellular structures that are sites for the production and storage of these compounds (Howe & Jander, 2008; De Geyter *et al*., 2012). In Arabidopsis leaves, wound‐inducible and JA‐inducible ER bodies are recognised as dynamic, organelle‐like structures that associate with specific defensive metabolites and proteins (Watanabe *et al*., 2013; Nakano *et al*., 2017; Nakazaki *et al*., 2019a). Among the proteins sequestered in ER bodies are β‐glucosidases, such as BGLU18 and PYK10/BGLU23, which hydrolyse indolic glucosinolates to toxic products (Nakano *et al*., 2017; Nakazaki *et al*., 2019a; Yamada *et al*., 2020). Previous work has shown that genes encoding many ER body‐associated proteins, including several BGLUs, are upregulated in *jazD* leaves (Guo *et al*., 2018b). A key finding of our study was that BLU18, which is a major protein component of iER bodies (Ogasawara *et al*., 2009), overaccumulated in *jazD* leaves coincident with the formation of ER bodies. Importantly, BGLU18 accumulation and ER body proliferation in *jazD* was dependent on MYC2/3/4. These findings confirmed and extended recent work showing that a *myc2 myc3 myc4* triple mutation blocks the accumulation of iER bodies in response to exogenous JA (Stefanik *et al*., 2019) and that MYC2/3 bind to the promoters of several ER body‐associated genes (Zander *et al*., 2020). Our finding that the JAZ–MYC regulon exerts more stringent control over iER than cER body genes agrees with previous reports showing that exogenous JA induces stronger expression of iER body genes (i.e. *TSA1*) relative to cER body genes (i.e. *PYK10*) (Geem *et al*., 2019), and supports the observed regulatory divergence between these two types of ER body (Stefanik *et al*., 2019).

Increasing evidence supports the hypothesis that ER bodies serve physiological functions related to glucosinolate‐based resistance to insect herbivory (Nakano *et al*., 2017; Nakazaki *et al*., 2019a; Rufian *et al*., 2021). In contrast with the canonical two‐component ‘mustard oil bomb’ system in which glucosinolates and their hydrolytic myrosinases are spatially separated in different cell types, ER bodies are thought to function as a single‐cell defence system in which ER body‐localised enzymes, such as BGLU18, act on glucosinolates released from vacuolar stores in response to cell disruption during herbivory (Nakano *et al*., 2014; Yamada *et al*., 2020). In keeping with this view, we demonstrated that the biosynthesis of indolic glucosinolates and the accumulation of ER bodies were coordinated by the JAZ–MYC signalling module and that the induction of these components in *jazD* is correlated with strong resistance to insect herbivory. ER bodies therefore provide an example of a multicomponent defence system that was recruited into the conserved JA–JAZ–MYC signalling module (Campos *et al*., 2014). Our finding that WT, *mycT*, *jazD* and *jazD mycT* leaves have markedly distinct glucosinolate profiles (Fig. 7a) further highlights the central role of JAZ–MYC interactions in controlling this layer of JA‐mediated leaf defence.

### Control of tryptophan metabolism by the JAZ–MYC module

A gap in our understanding of the growth–defence balance is how primary metabolism, as the engine of growth, is coordinated with the acute demand for defence compounds whose biosynthesis draws from amino acid, carbohydrate and other pools of primary metabolites (Aharoni & Galili, 2011; Guo *et al*., 2018a). The multifaceted role of amino acids in defence is exemplified by tryptophan, which as the most energetically costly amino acid is a precursor of defence compounds such as glucosinolates, camalexin, and various defence proteins (e.g. defensins) (Akashi & Gojobori, 2002; Tzin & Galili, 2010; Maeda & Dudareva, 2012; Barco & Clay, 2019). Interestingly, tryptophan biosynthetic capacity is upregulated by the JA pathway in Arabidopsis and other plant species (Bohlmann *et al*., 1996; Memelink *et al*., 2001; Dombrecht *et al*., 2007; Guo *et al*., 2018b). Here, we provide evidence that the JAZ–MYC module integrates the induced biosynthesis of defensive indolic compounds with requisite changes in tryptophan metabolism. Our finding that *mycT* is epistatic to *jazD* for both elevated tryptophan levels and increased sensitivity to 5‐MT indicates that MYC TFs boost tryptophan biosynthetic capacity, presumably to meet the demand for increased production of tryptophan‐derived defence compounds. Consistent with the elevated level of mRNAs encoding several tryptophan biosynthetic enzymes in *jazD* leaves (Guo *et al*., 2018b), persistent activation of JA signalling by coronatine treatment also increased the levels of these transcripts in WT plants (Fig. S4). Moreover, multiomic studies showed that MYC TFs bound directly to the promoter region of specific gene family members in the tryptophan and shikimate pathways (Zander *et al*., 2020). For example, among the three Arabidopsis genes (*DHS1‐3*) encoding 3‐deoxy‐d‐*arabino*‐heptulosonate 7‐phosphate synthase, which catalyses the initial step into the shikimate pathway, *DHS1* is the only member upregulated in *jazD* and is also the sole paralogue bound by MYC2/3 (Guo *et al*., 2018b; Zander *et al*., 2020). The catalytic properties and expression pattern of *DHS1* are consistent with a specialised role in leaf responses to stress (Yokoyama *et al*., 2021). These properties of *DHS1* and other JA‐regulated shikimate pathway genes support the general view that genes in general metabolism have been recruited into the JA signalling network in response to selective pressure to increase the production of defence compounds, without affecting the homeostasis of primary metabolism (Bohlmann *et al*., 1996; Pichersky & Lewinsohn, 2011; Guo *et al*., 2018a; Yokoyama *et al*., 2021). Our finding that increased rates of cellular respiration in *jazD* leaves depend on MYCs is also consistent with the notion that these TFs help to meet the increased metabolic demands of plant immune responses (Bolton, 2009; Guo *et al*., 2018b).

Our results support the view that growth penalties associated with JA responses result from active transcriptional reprogramming rather than passive (i.e. zero‐sum game) effects of reallocating primary metabolite pools to defence at the expense of growth (Campos *et al*., 2016; Kliebenstein, 2016; Guo *et al*., 2018a; Ballaré & Austin, 2019; Monson *et al*., 2021). Two general hypotheses may explain how MYC TFs negatively impact growth and reproductive output (Fig. 1a). First, MYCs may directly downregulate the expression of growth‐related genes, including genes involved in cell cycle control, photosynthesis and cell wall biogenesis (Attaran *et al*., 2014; Bömer *et al*., 2018; Zander *et al*., 2020). Although MYC2 and MYC3 have the potential to regulate over 20% of all genes in Arabidopsis, it is noteworthy that these TFs are predominately involved in transcriptional activation (Zander *et al*., 2020). An alternative hypothesis is that MYC‐mediated changes in metabolism generate metabolic imbalances that are perceived by cellular sensors such as target of rapamycin (TOR) or Snf1‐related protein kinase 1 (SnRK1), which in turn signal compensatory adjustments in growth rate (Smith & Stitt, 2007; Baena‐González & Hanson, 2017; Guo *et al*., 2018a). The blurred distinction between primary and secondary metabolism (Erb & Kliebenstein, 2020) suggests that metabolic sensors may include receptors for hormones or other metabolic signals that remain to be discovered. For example, the upregulation of the shikimate and tryptophan biosynthetic pathways by MYC TFs has the potential to alter the homeostasis of the growth hormone auxin, which is synthesised from tryptophan (Fig. 8a) (Mashiguchi *et al*., 2011). A future challenge in understanding the antagonistic relationship between plant immunity and growth or reproduction will be to determine how specific metabolic signals are generated and perceived during growth‐to‐defence transitions.

## Author contributions

QG, ITM and GAH designed the research; QG, ITM and GK performed the experiments; QG, ITM and GAH analysed the data; QG, ITM and GAH wrote the article. QG and ITM contributed equally to this work.

## Supporting information


**Fig. S1** Construction of the *jazD mycT* tredecuple mutant.
**Fig. S2** Identification using LC–MS/MS of proteins in the 65‐kDa band shown in Fig. 4.
**Fig. S3** Control of aliphatic glucosinolate accumulation by the JAZ–MYC regulon.
**Fig. S4** Expression of tryptophan biosynthesis genes is increased by persistent activation of JA signalling in wild‐type plants.
**Table S1** Mutant alleles used for construction of the *jazD mycT* line.
**Table S2** Oligonucleotide primers used for genotyping *jaz* and *myc* mutants.
Please note: Wiley Blackwell are not responsible for the content or functionality of any Supporting Information supplied by the authors. Any queries (other than missing material) should be directed to the *New Phytologist* Central Office.

## Data Availability

All data are included in the main article or in the supporting information.
