# Supplementary material for: MYC transcription factors coordinate tryptophan‐dependent defence responses and compromise seed yield in Arabidopsis
Source: New Phytol. 2022 Jun 21;236(1):132–45. doi: 10.1111/nph.18293 (PMC9541860; doi:10.1111/nph.18293)
Supplement: Supplementary file 1 — Fig. S1 Construction of the jazD mycT tredecuple mutant. Fig. S2 Identification using LC–MS/MS of proteins in the 65‐kDa band shown in Fig. 4. Fig. S3 Control of aliphatic glucosinolate accumulation by the JAZ–MYC regulon. Fig. S4 Expression of tryptophan biosynthesis genes is increased by persistent activation of JA signalling in wild‐type plants. Table S1 Mutant alleles used for construction of the jazD mycT line. Table S2 Oligonucleotide primers used for genotyping jaz and myc mutants. Please note: Wiley Blackwell are not responsible for the content or functionality of any Supporting Information supplied by the authors. Any queries (other than missing material) should be directed to the New Phytologist Central Office. [file NPH-236-132-s001.pdf]

***New Phytologist* Supporting Information**

Article title: MYC transcription factors coordinate tryptophan-dependent defense responses and compromise seed yield in Arabidopsis

Authors: Qiang Guo, Ian T. Major, George Kapali, Gregg A. Howe

Article acceptance date: 29 May 2022

The following Supporting Information is available for this article:

Supplemental Fig. S1 Construction of the *jazD mycT* tredecuple mutant of Arabidopsis.
Supplemental Fig. S2 Identification by LC-MS/MS of Arabidopsis proteins in the 65-kD band shown in Figure 4.
Supplemental Fig. S3 Control of aliphatic glucosinolate accumulation by the JAZ-MYC regulon in Arabidopsis.
Supplemental Fig. S4 Expression of tryptophan biosynthesis genes is increased by persistent activation of JA signaling in wild-type Arabidopsis.
Table S1 Mutant alleles used for construction of the *jazD mycT* line of Arabidopsis.
Table S2 Oligonucleotide primers used for genotyping *jaz* and *myc* mutants of Arabidopsis.

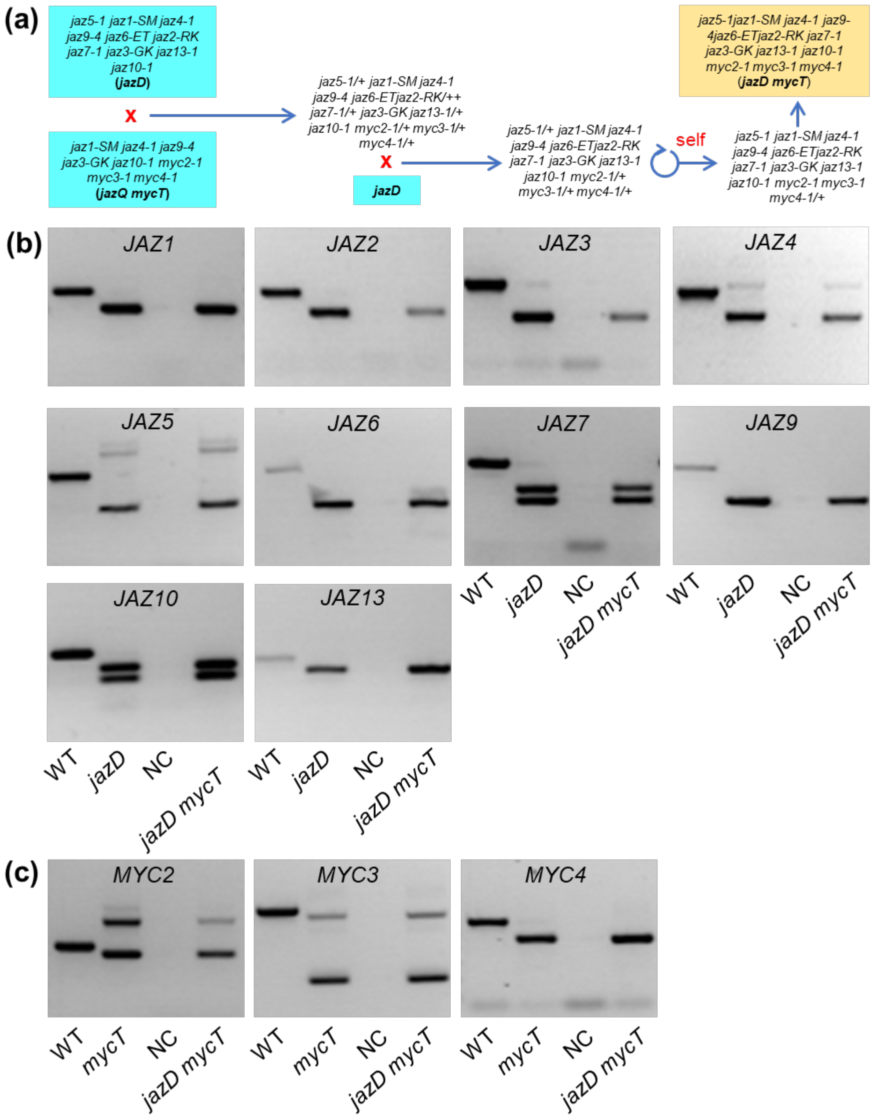

Fig. S1 Construction of the *jazD mycT* tredecuple mutant of Arabidopsis. (a) Pedigree used for construction of *jazD mycT.* Red 'x' denotes cross-pollination and red 'self' denotes self-pollination. Blue arrows indicate one generation. Blue-shaded genotypes have been reported previously (Major et al., 2017; Guo et al., 2018). The tredecuple *jazD mycT* line was identified by PCR-based genotyping of progeny from the indicated segregating parental line. (b, c) Genotyping of *jazD mycT* for *JAZ* (b) and *MYC* (c) loci was performed using primer sets flanking DNA insertion sites and a third primer recognizing the T-DNA border (Supplemental Table S2). NC, no template control.

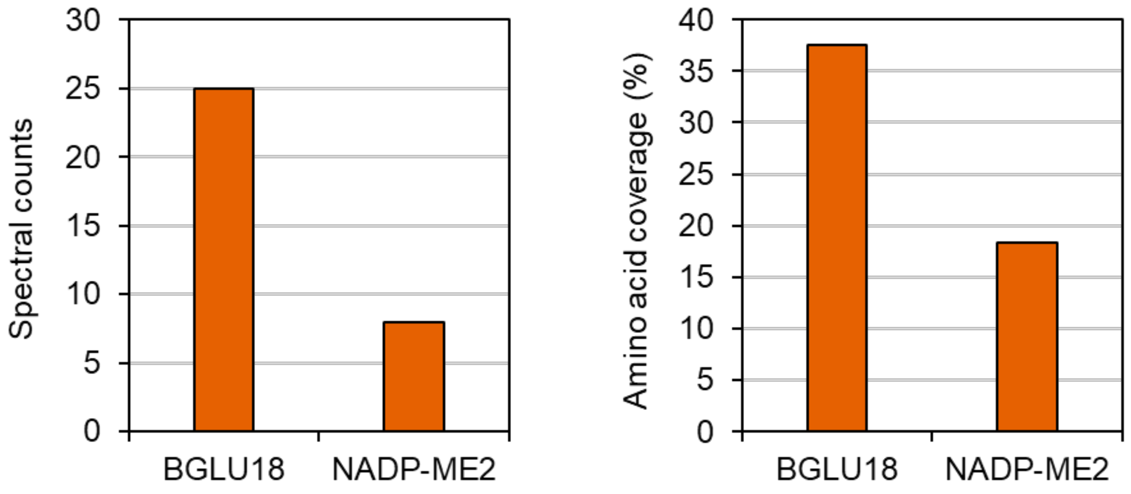

Fig. S2 Identification by LC-MS/MS of Arabidopsis proteins in the 65-kD band shown in Figure 4. The number of spectral counts (left) and percent amino-acid coverage of the identified proteins (right) are shown. BGLU18 (β-glucosidase 18, AT1G52400) and NADP-ME2 (NADP-malic enzyme 2, AT5G11670) were the only *jazD*-specific proteins having at least two spectral counts and >5% total amino acid coverage. A corresponding gel slice from the WT sample was analyzed in parallel by LC-MS/MS as a control. Peptides corresponding to BGLU18 and NADP-ME2 were not identified in the control sample.

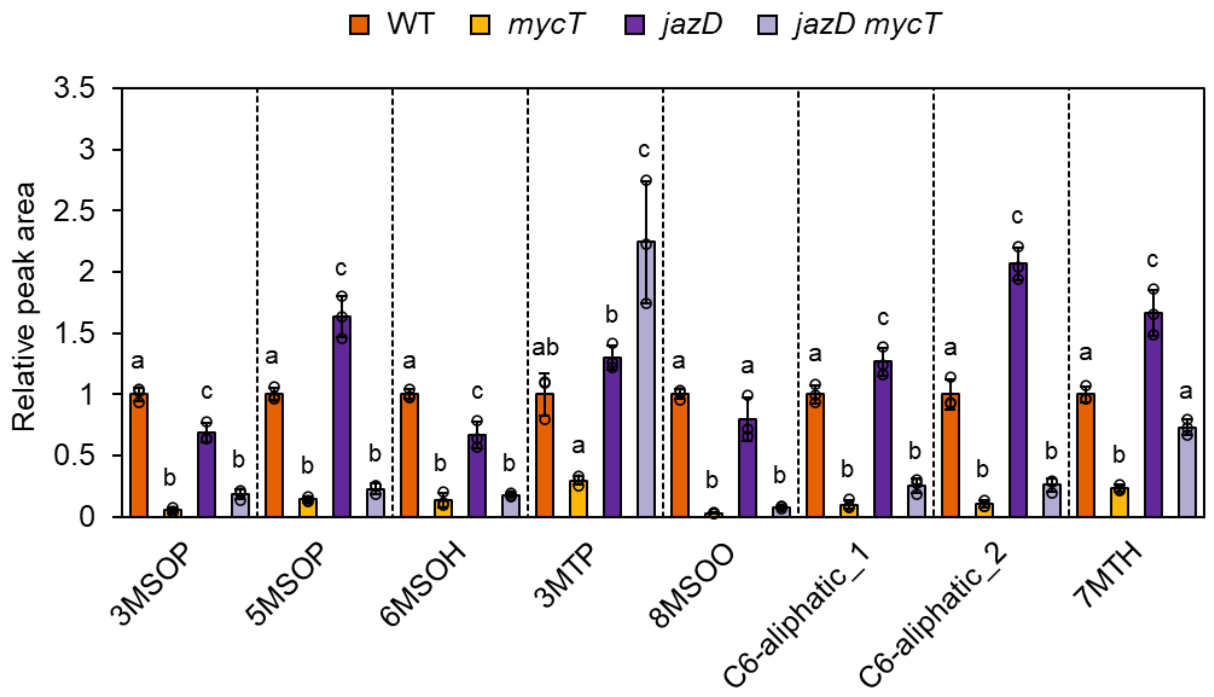

Fig. S3 Control of aliphatic glucosinolate accumulation by the JAZ-MYC regulon in Arabidopsis. Level of eight aliphatic glucosinolates were measured in leaves of 26-day-old WT, *mycT*, *jazD*, and *jazD mycT* plants. Glucosinolates were extracted from leaves and quantified by LC-MS. Values show the mean +/- SD (n=3 biological replicates). The peak area from LC-MS for the indicated compound in the WT sample was set to "1" and the peak area of the same compound in other genotypes was scaled to the WT sample. Letters denote significant differences according to Tukey's HSD test ($P < 0.05$). 3MSOP, 3-methylsulphinylpropyl (glucoiberin); 5MSOP, 5-methylsulphinylpentyl (glucoalyssin); 6MSOH, 6-methylsulphinylhexyl (glucohesperin); 3MTP, 3-methylthiopropyl (glucoiberverin); 8MSOO, 8-methylsulphinyloctyl (glucohirsutin); 7MTH, 7-methylthioheptyl.

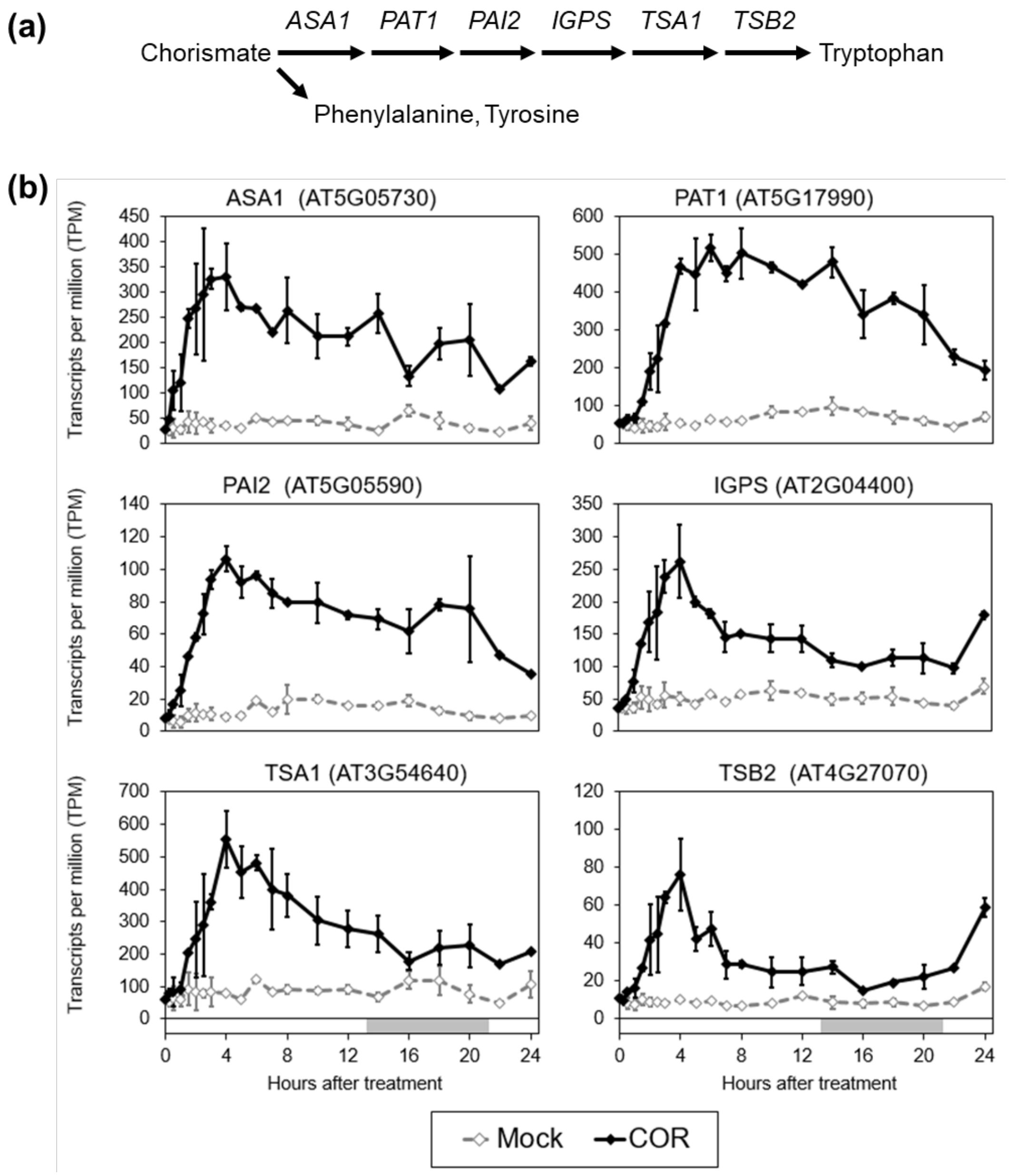

Fig. S4 Expression of tryptophan biosynthesis genes is increased by persistent activation of JA signaling in wild-type Arabidopsis. (a) Simplified pathway for tryptophan biosynthesis. (b) RNA-seq expression levels (transcripts per million, TPM) of tryptophan biosynthesis genes (*ASA1*, *PAT1*, *PAI2*, *IGPS*, *TSA1*, and *TSB2*) at various times after treatment of Col-0 seedlings with coronatine (filled, black points) or a mock control (open, gray points). Data points indicate the mean expression levels +/- SD. Photoperiod is denoted above the x-axis. RNA-seq data are from Attaran *et al.* (2014).

Table S1 Mutant alleles used for construction of the *jazD mycT* line of Arabidopsis.

Mutant	Original name	Source	Accession	Mutagen	Resistance[a]
jaz1-2	SM_3.22668	JIC SM	Col-0	dSpm transposon	Basta[1]
jaz2-3	RIKEN_13-5433-1	RIKEN	No-0	*Ds* transposon	Hygromycin[1]
jaz3-4	GK-097F09	GABI Kat	Col-0	T-DNA (pAC161)	Sulfadiazine[1]
jaz4-1	SALK_141628	SALK	Col-0	T-DNA (pROK2)	Kanamycin[2]
jaz5-1	SALK_053775	SALK	Col-0	T-DNA (pROK2)	Kanamycin[1]
jaz6-4	CSHL_ET30	CSHL	Ler	*Ds* transposon (Enhancer trap GUS)	Kanamycin[1]
jaz7-1	WiscDsLox7H11	Wisconsin	Col-0	T-DNA (pWiscDsLox)	Basta[3]
jaz9-4	GK_265H05	GABI Kat	Col-0	T-DNA (pAC161)	Sulfadiazine[1]
jaz10-1	SAIL_92_D08	SAIL	Col-0	T-DNA (pCSA110)	Basta[1], GUS
jaz13-1	GK_193G07	GABI Kat	Col-0	T-DNA (pAC161)	Sulfadiazine[3]
myc2-1	SALK_040500	SALK	Col-0	T-DNA (pROK2)	Kanamycin[2]
myc3-1	GK-445B11	GABI Kat	Col-0	T-DNA (pAC161)	Sulfadiazine[1]
myc4-1	GK-491E10	GABI Kat	Col-0	T-DNA (pAC161)	Sulfadiazine[1]

[a]Resistance of the mutant line to the indicated selectable marker was (1) confirmed, was (2) silenced or was (3) not tested.

Table S2 Oligonucleotide primers used for genotyping *jaz* and *myc* mutants of Arabidopsis.

Gene	Locus	Primer	Sequence (5'-3')
JAZ1	AT1G19180	JAZ1_F	ACCGAGACACATTCCCGATT
		JAZ1_R	CATCAGGCTTGCATGCCATT
		JAZ1_border	ACGAATAAGAGCGTCCATTTTAGAG
JAZ2	AT1G74950	JAZ2_F	TCTTCCTCGTGACAAAACGCA
		JAZ2_R	CCAAACACAGAACCATCTCCACA
		JAZ2_border	CCGGATCGTATCGGTTTTCG
JAZ3	AT3G17860	JAZ3_F	ACGGTTCCTCTATGCCTCAAGTC
		JAZ3_R	GTGGAGTGGTCTAAAGCAACCTTC
		JAZ3_border	ATAACGCTGCGGACATCTACATT
JAZ4	AT1G48500	JAZ4_F	TCAGGAAGACAGAGTGTTCCC
		JAZ4_R	TGCGTTTCTCTAAGAACCGAG
		JAZ4_border	TTGGGTGATGGTTCACGTAG
JAZ5	AT1G17380	JAZ5_F	GCTTATACCGAAAACCCGATTCCAG
		JAZ5_R	GGCTCATTGAGATCAGGAAGAACCA

		JAZ5_border	TTGGGTGATGGTTCACGTAG
JAZ6	AT1G72450	JAZ6_F	GACACACATCACTGTCACTTC
		JAZ6_R	AGTTTCTGAGGTCTCTACCTTC
		JAZ6_border	CCGTTTTGTATATCCCGTTTCCGT
JAZ7	AT2G34600	JAZ7_F	ATGCGACTTGGAACTTCGCC
		JAZ7_R	GGAGGATCCGAACCGTCTG
		JAZ7_border	ACGTCCGCAATGTGTTATTA
JAZ9	AT1G70700	JAZ9_F	TACCGCATAATCATGGTCGTC
		JAZ9_R	TCATGCTCATTGCATTAGTCG
		JAZ9_border	CTTTGAAGACGTGGTTGGAACG
JAZ10	AT5G13220	JAZ10_F	ATTTCTCGATCGCCGTCGTAGT-3
		JAZ10_R	GCCAAAGAGCTTTGGTCTTAGAGTG
		JAZ10_border	GTCTAAGCGTCAATTTGTTTACACC
JAZ13	AT3G22275	JAZ13_F	GCACGTGACCAAATTTGCAGA
		JAZ13_R	TGAAGAGAGGAGGATGATGAGGA
		JAZ13_border	AAACCTCCTCGGATTCCATTGC

MYC2	AT1G32640	MYC2_F	GCTACAACCAACGATGAATC
		MYC2_R	TCATCAACAGCGTCATCCGA
		MYC2_border	TTGGGTGATGGTTCACGTAG
MYC3	AT5G46760	MYC3_F	GTTAGATCAGCTGCGAATGATTCGG
		MYC3_R	CTCCGACTTTCGTCATCAAAGCAAC
		MYC3_border	ATAACGCTGCGGACATCTACATT
MYC4	AT4G17880	MYC4_F	GGATCCATGTCTCCGACGAATGTTCAAGTA
		MYC4_R	TCTCTCACAACTTGATCCAGCTAA
		MYC4_border	ATAACGCTGCGGACATCTACATT

References

Attaran E, Major IT, Cruz JA, Rosa BA, Koo AJK, Chen J, Kramer DM, He SY, Howe GA. 2014. Temporal dynamics of growth and photosynthesis suppression in response to jasmonate signaling. *Plant Physiology* **165**: 1302-1314.

Guo Q, Yoshida Y, Major IT, Wang K, Sugimoto K, Kapali G, Havko NE, Benning C, Howe GA. 2018. JAZ repressors of metabolic defense promote growth and reproductive fitness in Arabidopsis. *Proceedings of the National Academy of Sciences, USA* **115**: E10768-E10777.

Major IT, Yoshida Y, Campos ML, Kapali G, Xin XF, Sugimoto K, de Oliveira Ferreira D, He SY, Howe GA. 2017. Regulation of growth-defense balance by the JASMONATE ZIM-DOMAIN (JAZ)-MYC transcriptional module. *New Phytologist* **215**: 1533-1547.
